# Pulmonary lymphangiomatosis: insights into an ultra-rare disease

**DOI:** 10.1186/s12931-024-03040-5

**Published:** 2024-11-26

**Authors:** M. Polke, N. Polke, S. Piel, E. Brunnemer, J. Wälscher, K. Buschulte, A. Warth, C. P. Heussel, M. Eichinger, L. Frankenstein, M. Eichhorn, S. Miliauskas, F. J. F. Herth, M. Kreuter

**Affiliations:** 1 Center for Interstitial and Rare Lung Diseases, Pneumology and Critical Care Medicine, Thoraxklinik, University of Heidelberg, Heidelberg, Germany; 2 Translational Lung Research Center Heidelberg, Member of the German Center for Lung Research DZL, Heidelberg, Germany; 3 Member of ERN-LUNG, Heidelberg, Germany; 4 Center for Interstitial and Rare Lung Diseases, Pneumology Department, Ruhrlandklinik, University Hospital, University of Essen, Essen, Germany; 5 Institute of Pathology, University of Heidelberg, Heidelberg, Germany; 6 Dagnostic and Interventional Radiology with Nuclear Medicine, Thoraxklinik, University of Heidelberg, Heidelberg, Germany; 7 Department of Cardiology, University of Heidelberg, Heidelberg, Germany; 8 Department of Thoracic Surgery, University of Heidelberg, Heidelberg, Germany; 9 Department of Pulmonology, Lithuanian University of Health Sciences, Kaunas, Lithuania; 10 Center for Pulmonary Medicine, Department of Pneumology, Mainz University Medical Center, Mainz, Germany; 11 Center for Pulmonary Medicine, Department of Pulmonary, Critical Care & Sleep Medicine, Marienhaus Clinic Mainz, Mainz, Germany

## Abstract

**Background:**

Pulmonary lymphangiomatosis (PL) is an ultrarare disease characterized by diffuse infiltration of the lung, pleura and/or mediastinum by abnormal lymphatic proliferation. Consented diagnostic or treatment approaches are not established. We therefore aimed to collect data on diagnostics and treatments in a cohort of patients with PL from a tertiary center for rare lung diseases.

**Methods:**

Clinical, radiological and outcome data from PL patients were collected retrospectively.

**Results:**

12 patients were diagnosed between 1996 and 2022 in our center. PL was diagnosed more commonly in female (58%), never smokers (75%) and younger patients (mean age 42 years). Main clinical symptoms comprised haem- and chyloptysis (58%) and dyspnea on exertion (83%). Pulmonary function was mostly restrictive (mean VC 59%) with impaired DLCO (mean 65%). Radiological assessment mainly showed mediastinal involvement (83%), and pleural effusion (67%), pleural thickening (67%) and bronchial wall thickening (67%) while interstitial changes were rare. Diagnosis was confirmed by surgical or transbronchial cryobiopsy. 8 patients were treated with sirolimus, 3 of these combined with a surgical intervention and in one case surgical intervention was necessary 9 months after initiation of sirolimus. Clinical and radiological improvement was demonstrated for all patients treated with sirolimus. 1 patient received a lung transplant due disease progression. Survival rates were 90% after a mean follow up of at least 3 months.

**Conclusion:**

This case series illustrates the variability of the clinical presentation of PL. Among our patients, those treated with sirolimus showed significant clinical, functional and radiological improvement. However, further investigation is needed to understand the pathogenesis of lymphangiomatosis in order to establish therapeutic approaches.

**Supplementary Information:**

The online version contains supplementary material available at 10.1186/s12931-024-03040-5.

## Background

Pulmonary lymphangiomatosis is an ultrarare disease that usually occurs in childhood and adulthood. The term ultrarare was initially introduced by the by the National Institute for Health and Care Excellence for drugs with indication for diseases that have a prevalence < 1 per 50.000 persons [1], however it is not legally defined.

PL is postulated to be congenital and to affect both sexes equally. Symptoms are variable including asymptomatic to severely respiratory distressed patients leading to death [2]. Most patients present with dyspnea on exertion, haemoptysis, chylous effusion or chest pain [3]. Pulmonary function tests can show both obstructive and restrictive ventilation disorders in addition to respiratory failure [4]. Computed tomography (CT) of the lungs can show pleural thickening, pleural effusions, septal and peribronchovascular thickening as well as mediastinal soft tissue infiltration [5]. Biopsy is usually obtained by video-assisted thoracoscopy or transbronchial biopsies [6]. However, most cases within the literature were diagnosed by thoracoscopic wedge resection. Histologically, lymphangiomas with lymphoid endothelial cells are positive for CD-31 and D2-40 [7]. Vascular endothelial growth factor (VEGF)-D is an established lymphangiogenic factor [8] probably playing an important role in the pathogenesis of lymphangiomatosis [9]. There is currently no established treatment regimen and no causal therapy. Current treatments aim to reduce increased lymph secretion. Case reports showed some effectiveness for a number of drugs including the mTOR inhibitor sirolimus, the unselective beta-blocker propranolol, chemotherapeutic as well as surgical treatments and radiotherapy [2, 9].

## Methods

We analysed our database for patients with rare lung diseases diagnosed between 1996–2022 for confirmed pulmonary lymphangiomatosis. Initial CTs from identified cases were obtained and re-reviewed by an experienced thoracic radiologist. Demographic variables (age; sex; dyspnoea; cough; chest pain; smoking status), pulmonary function tests, diffusing capacity of the lung for carbon monoxide (DLCO); histopathological patterns and forms of treatments as well as outcomes were collected.

We also conducted a literature research on the PubMed Central® (PMC) to gain an estimated number of case reports on pulmonary lymphangiomatosis and pulmonary involvement in lymphangiomatosis respectively over the last 40 years. The search term used was “pulmonary lymphangiomatosis”. All results were reviewed critically to identify relevant reports.

## Results

During 1996 to 2022 twelve patients were diagnosed with pulmonary lymphangiomatosis in our tertiary ILD center. Two of these were diagnosed with probable pulmonary lymphangiomatosis due to inconclusive histology. Pulmonary lymphangiomatosis was diagnosed more commonly in female (58%), never smokers (75%) and younger patients (mean age 42 years). Main clinical symptoms comprised haem- and chyloptysis (58%) and dyspnea on exertion (83%). Two patients suffered both from haemoptysis and chyloptysis, and one patient suffered from ventilatory failure. Pulmonary function was restrictive (mean VC 59%) with impaired DLCO (mean 65%) in most cases (Table 1).

**Table 1:** Patients’ characteristics at first diagnosis

Case	Patient 1	Patient 2	Patient 3	Patient 4	Patient 5	Patient 6	Patient 7	Patient 8	Patient 9	Patient 10	Patient 11	Patient 12
Age at diagnosis	42	13	37	29	27	63	44	55	53	59	27	52
Sex	Female	Female	Female	Male	Female	Female	Female	Male	Female	Male	Male	Male
Symptoms	Chyloptysis, haemoptysis	Dyspnoea, cough, haemoptysis	Dyspnea, fatigue	Chyloptysish, haemoptysis, night sweating, loss of weight, pericarditis	Dyspnea, chyloptysis, fatigue, night sweating	Dyspnea, chyloptysis, pneumothorax	Dyspnea, haemoptysis, cough	Dyspnea	Dyspnea, cough, white sputum, join pain	Dyspnea, cough	Hemoptysis, dyspnea, night sweats, chylous fluid obtained during thoracenteses	Dyspnea, thoracic pain while breathing
Smoking Status	Non-smoker	Non-smoker	6 py (ex-smoker)	16 py (ex-smoker)	Non-smoker	Non-smoker	Non-smoker	Non-smoker	Non-smoker	Non-smoker	4 pack-years (ex-smoker)	Non-Smoker
Comorbidities	Autoimmun-Thyreoiditis	None	Melanoma, arterial hypertension, hyperlipidemia	Right heart insufficiency, liver cysts, nephrolithiasis, cholecystolithiasis	None	Pulmonary embolism, arterial hypertension, chronic kidney failure, thyroid hypofunction, chronic pain syndrome	None	Coronary calcifications, cholecystolithiasis, arterial hypertension	Asthma, diabetes mellitus 2, thyroid hypofunction, arterial hypertension, hepatic steatosis	Arterial hypertension, depression, factor V Leiden mutation, spinal stenosis, spondylarthrosis, degenerative scoliosis, sulcus ulnaris syndrome, coxarthrosis, gonarthrosis	Pulmonary embolism, sinus tachycardia, autoimmune thyroiditis	Littoral cell angioma, thyroid hypofunction, arterial hypertension
Medication at diagnosis	Thyroxine, citaloprame, oral contraceptive	None	None	Digoxine,torasemide, metoprolol, pantoprazol, oxycodone, morphin	Inhalative beclometasone/formoterole, pantoprazole, thyroxine	Furosemide, spironolacton, pantoprazole, tilidin	Pantoprazole, hydrochlorothiazide, potassium	Bisoprolol, torasemide, vitamin B complex	inhaled corticosteroid/long-acting beta-agonist, long-acting anticholinergics, salbutamol, thyroxine, dapagliflozin, sartan, hydrochlorothiazide, ibuprofen, magnesium	None	Rivaroxaban, methylprednisolone, metoprolol	Thyroxine, sartan
FVC (l/%) at baseline	3.46/99	0.89/24	3.62/102	1.34/26	3.62/93	–	1.30/36	-/60	2.62/83	1.94/36	2.87/43	2.35/46
FEV1 (l/%) at baseline	2.29/76	0.814/26	3.01/99	1.23/30	2.74/81	–	1.26/43	2.41/62	2.30/91	1.63/40	2.40/44	1.56/39
FEV1%FVC (%) at baseline	63	92	84	92	76	–	96	82	88	81	84	65
DLCO SB (mmol/min/kPa/%) at baseline	6.54/72.7	n.a.	n.a.	n.a.	9.76/64	–	8.40/35	-/83	6.55/86	5.35/49	–	6.48/62
6MWD (m)	535	n.a	500	–	–	252	285	–	–	485	358	–
Histology	Bronchial vascular and subpleural ectatisis lymphatics intra-alveolar hemorrhages. Lymphatic vessels D2-40 -positive	Lymphangiomas with lymphoid endothelial cells. Lymphatic vessels D2-40 -positive	Fibromuscular soft tissue with endothelial lined cavities; CD34 and D2-40 positive	Benign lymphohematoid lesion mediastinal lymphangioma	Soft tissue with lymphangioma	Soft tissue with lymphangioma	Dilated lymphatic vessels (D2-40, CD31, CD34 positive) besides lymphoidcellaggregation	No evidence for lymphangiectasia or lymphangiomatosis	No evidence for lymphangiectasia or lymphangiomatosis	Conspicuous vessel architecture (CD31 and D2-40 positive)	H&E-stained and D2-40 immunohistochemistry of lung tissue showing dilated lymphatic vessels, secondary changes, including thickened pleura, bullous alveoli and organizing pneumonia	Dilated lymphatic vessels (D2-40 positive)
Diagnostic procedure	Surgical lung biopsy (SLB): 1 sample (wedge biopsy), from diseased, sample size 6 × 3x2cm	SLB: 2 wedge biopsies, from diseased, sample size 12.5 × 9.3x6.5 cm and 13.8 × 4.2x4.1 cm	SLB: 1 sample from mediastinal tumor + pericard + thymus, from diseased, max. sample size 4 × 3x1cm	SLB: samples from pleura, from diseased, max. sample size 17 × 112x4cm	SLB: 1 wedge biopsy and samples from mediastinum + pericard, from diseased, sample size max. 4.3 × 1.3x1.3 cm	SLB: 1 from mediastinal tumor, from diseased, sample size 7.5 × 3.7x1cm	Transbronchial cryo-biopsy (TBCB): 4 samples, from diseased, sample size max. 0.6 cm	SLB: 1 sample from pleura, random location, sample size 2.5 × 1.8x0.8 cm	Endo bronchial biopsy (EBB): 2 samples, from diseased, sample size max. 0.3 cm	EBB: 5 samples from 2 different locations, from diseased, sample size max. 1,2 cm	SLB: 1 atypical resection, from diseased, sample size 9 × 2.8x2.4 cm	EBB: 1 sample, from diseased, sample size max. 1.3 cm
VEGF-D (ng/ml)	–	–	–	–	0.390	–	–	0.407	–	0.248	–	0.019

**Fig. 1:**
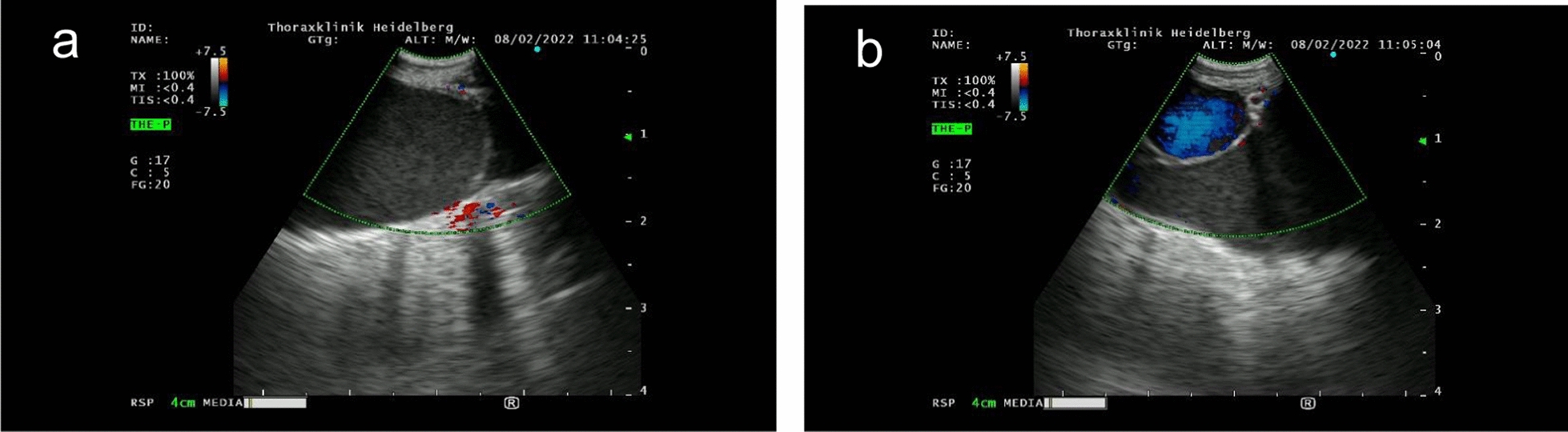
1a and 1b: Endobronchial ultrasound of mediastinal mass showing dilated low-flow non-blood vessels

**Fig. 2:**
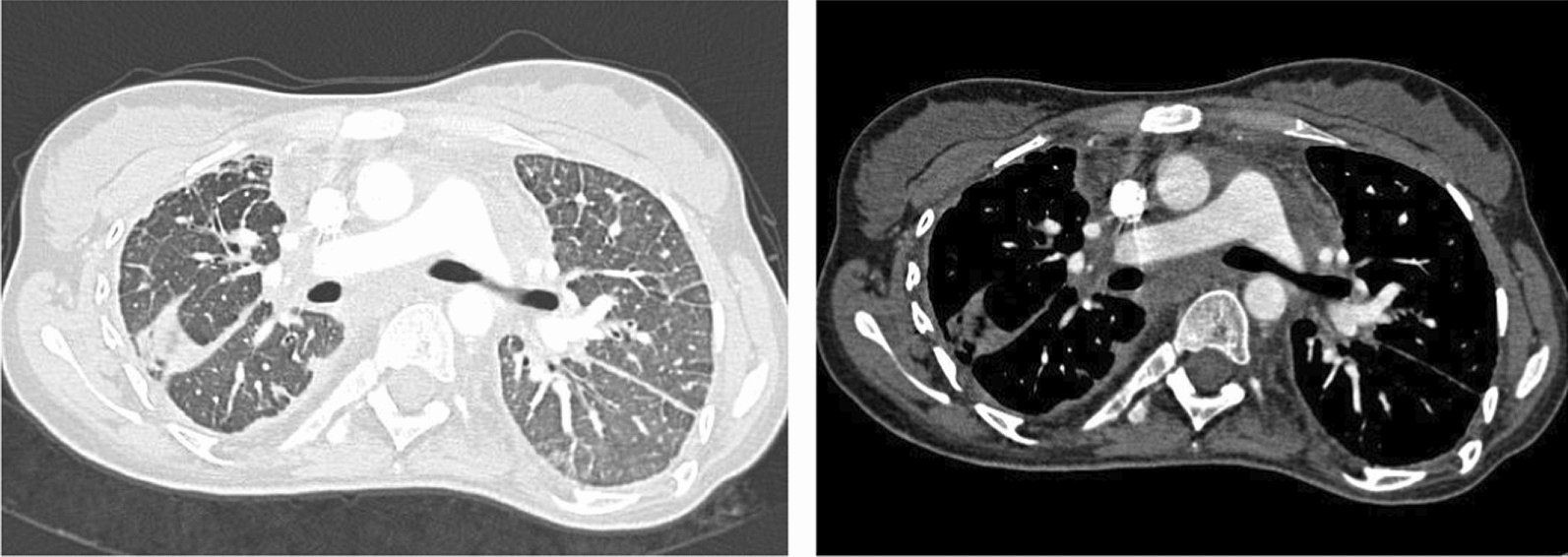
CT-Thorax: mediastinal mass, pleural thickening and septal thickening in PL

**Fig. 3:**
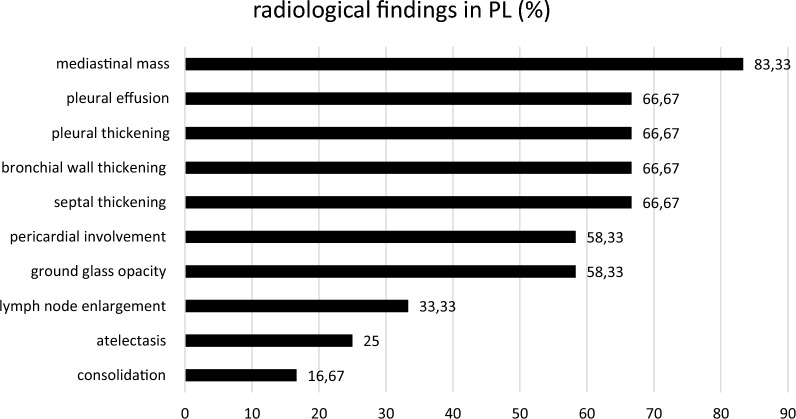
Radiological findings in PL and their incidence

Radiological assessment showed mediastinal involvement in all patients (Figs. 1a and b, 2, 3) except of one. Pleural effusion, pleural thickening, bronchial wall thickening and septal thickening (Figs. 2, 3) were common radiological findings (each 67%). All radiological findings and their frequency are shown in Fig. 3.

Diagnosis was confirmed by surgical biopsy or transbronchial biopsy in all patients. Histology showed pleural and peribronchial vascular ectasia with lymphangioma, D2-40 positive, in most cases.

**Fig. 4:**
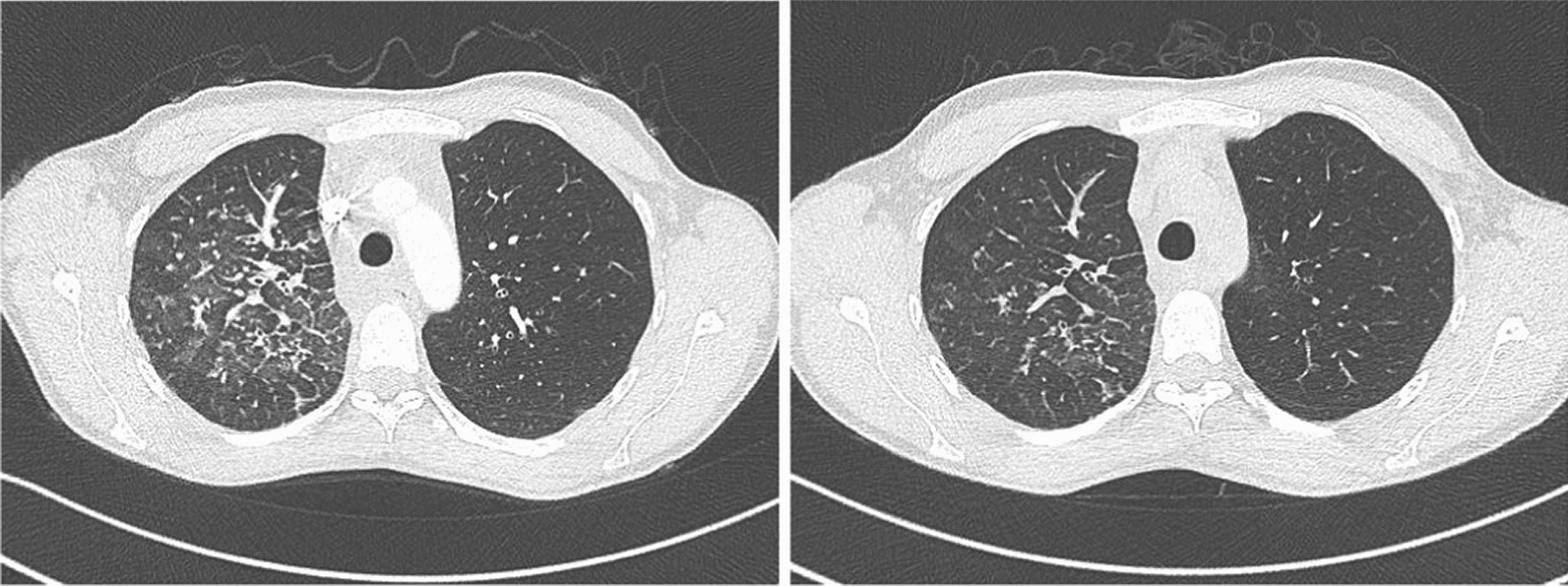
CT-Thorax before and after 3 months of treatment with sirolimus

9 patients were treated with sirolimus, 4 of these combined with a surgical intervention/resection. Clinical and/or radiological improvement was demonstrated for all patients treated with sirolimus for at least 3 months follow up (Table 2; Fig. 4).

**Table 2:** treatments and outcome

Case	Patient 1	Patient 2	Patient 3	Patient 4	Patient 5	Patient 6	Patient 7	Patient 8	Patient 9	Patient 10	Patient 11	Patient 12
Therapy	Sirolimus	NIV, lung-transplantation	Surgery with mediastinal cyst removal and thoracic duct ligation	Thoracoscopic pericardial fenestration, removal of the mediastinal tumor, Ductus thoracic ligature and talcum pleurodesis	Thoracoscopic pericardial fenestration, followed by sirolimus one months later which was paused after the patient became pregnant	Thoracoscopic talcum pleurodesis, thoracic duct ligation without success, followed by thoracic radiation due to persistent chylothorax and sirolimus	Sirolimus	Thoracoscopic talcum pleurodesis, thoracic radiation, sirolimus, oxygen therapy	Sirolimus	Sirolimus recommended	Sirolimus + propranolol only for 9 months Followed by pericardiectomy, adhesiolysis and talcum pleurodesis due to deterioration, continuation with sirolimus	Sirolimus
Outcome	Clinical and radiological improvement after 3 months of therapy with sirolimus, stable disease in the following 25 months, afterwards loss of follow up	Death 8 years after diagnosis due to a complication after lung transplantation which was performed due to a progressive disease	Loss of follow-up	1 month after surgery no symptoms anymore, afterwards loss of follow up	Improvement of pericardial effusion 12 months after sirolimus therapy, stable disease during pregnancy and time afterwards until now (another 26 months)	Clinical and radiological improvement and stabilization 6 months of sirolimus, treatment for 45 months, afterwards loss of follow up	Clinical and radiological improvement for 11 months after initiating sirolimus, loss of follow up after a treated infection	Regressive chylothorax after 3 months of sirolimus treatment, discontinuation due to phlebitis for one months, followed by reuptake, afterwards loss of follow up	Clinical improvement, discreet progression in CT scan (initiation of sirolimus was delayed to concerns of the patient in terms of adverse event and started after progression of symptoms)	Stable situation for 3 months, then loss of follow-up	Clinical and radiological improvement for approx. 3 months; after combining pharmacological and surgical treatments improvement of symptoms, lung function, X-ray findings and 6MWT	Stable clinical and radiological situation after 6 months

**Fig. 5:**
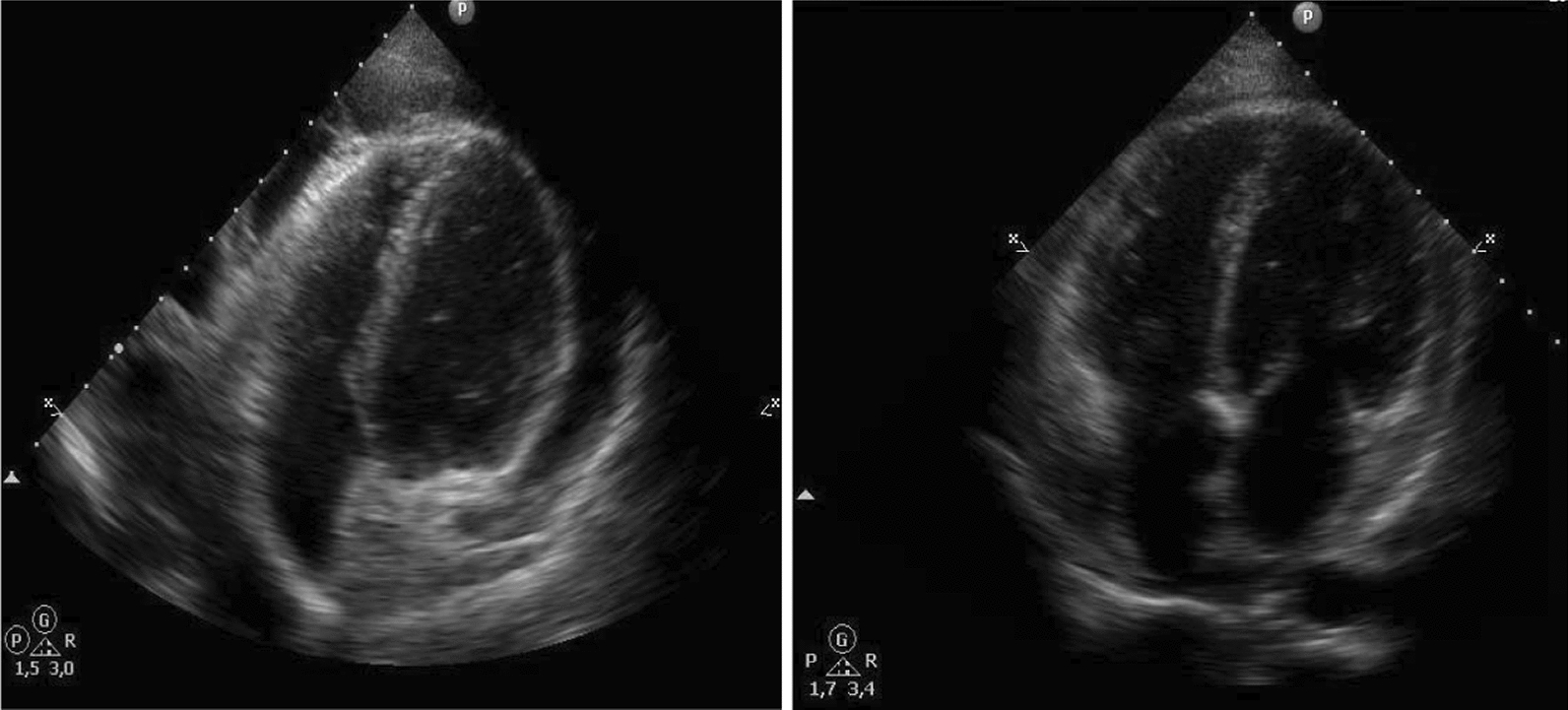
Echocardiography in a patient with PL and associated chylous pericardial effusion before and after 8 months of treatment with sirolimus

One patient showed an improvement of the PL associated chylous pericardial effusion due to the treatment with sirolimus (Fig. 5).

One of the patients who received sirolimus in combination with propranolol presented with a deterioration 9 months after initiation with progressive pleural effusion and symptoms of heart failure. Then, surgical intervention (adhesiolysis, talcum pleurodesis, pericardiectomy) was performed followed by a pharmacological treatment with sirolimus only. 3 months afterwards an improvement in clinic, lung function and in X-ray was accomplished. In 1 patient the initiation with sirolimus was planned but not started due to waiting for the approval of an off-label use and then loss of follow up. 1 patient received lung transplantation due to disease progression after initial surgical therapy but died just afterwards due to complication. The outcome for two patients could not be evaluated due to loss off follow-up. Survival rates were 92% after a mean follow up of at least 3 months (Table 2).

The estimated number of case reports on pulmonary lymphangiomatosis and pulmonary involvement of lymphangiomatosis respectively in Pubmed Central® (PMC) was 71 over the last 40 years (Supplement).

## Discussion

Pulmonary lymphangiomatosis is caused by proliferation of lymphatic vessels in soft tissue [2]. Our case series illustrates the variability of clinical presentation and affections of different sites of the thorax in pulmonary lymphangiomatosis. Because of the rarity of lymphangiomatosis, it is difficult to establish an evidence-based treatment strategy. Most treatments are supportive aiming to decompress adjacent structures and chylous fluid accumulation. Here, we give further insights into this ultrarare disease by adding more knowledge on diagnostics and therapeutic possibilities, especially under the treatment with sirolimus. Among our patients, those treated with sirolimus showed significant clinical, pulmonary and CT morphological improvement with a therapeutic level of 5 ng/ml. This is in line with a recent systematic review which reports that treatment with the mTOR inhibitor sirolimus was an effective and safe treatment for patients with complicated vascular anomalies including lymphangiomatosis that was refractory to other therapies [10]. As an underlying effect of sirolimus it is discussed that sirolimus binds to VEGF receptor 3 on the surface of the lymphatic endothelium [11]. Our data are also in line with some limited data from case reports confirming a successful treatment with sirolimus in several cases [12–15]. Reports on other therapies in PL are sparse. One potential treatment option might be radiation therapy by radiation-induced fibrosis of the lymphatic endothelium leading to destruction of the lymph vessels resulting in a regression of lesions for several months [16]. This therapy option was also chosen in one of the patients, where the combination of radiotherapy and sirolimus finally lead to a significant clinical improvement. Regarding surgical therapy, our data suggest that resection may have an effect for localized lung or mediastinal lesions. Other surgical treatments are pleurodesis, parietal pleurectomy and ligation of the thoracic duct [17]. However, disease manifestations might relapse after surgical procedures since remaining diseased tissue can lead to uncontrolled proliferation of lymphatic vessels. One case report illustrates a successful bilateral lung transplantation which underscores the importance of accurate selection of patients [18].

Other drugs such as bevacizumab or interferon alpha 2b seem to have a positive impact on the clinical course of the disease [19, 20].

In certain clinical cases sclerotherapy e.g. with doxycycline might be a therapeutic option [21].

Conservative treatment strategies such as medium-chain triglycerides and high-protein diets or total parenteral nutrition were not effective [22].

Vascular endothelial growth factor (VEGF)–D is an established growth factor for lymphangiogenesis, e.g. in lymphangioleiomyomatosis (LAM) [23]. Thus, this protein might be important as a therapeutic and/or diagnostic biomarker also in lymphangiomatosis. In 3 of the presented cases, serum levels of VEGF-D were useful for diagnosing pulmonary lymphangiomatosis. However, further investigation is needed to establish a cut-off for serum levels of VEGF-D as useful guidance for diagnostic and therapeutic approaches in this disease. As propranolol, a non-selective β-blocker, reduces the levels of VEGF-D, also this may be an alternative treatment option. In a case report of a child suffering from lymphangiomatosis, reduction of pleural effusion could be shown after the treatment with propranolol [9]. In one of our cases propranolol was used in combination with sirolimus and could stabilize disease progression but for 9 months only.

In the light of these considerations, we assume that sirolimus treatment is effective in pulmonary lymphangiomatosis. However, it is unclear if sirolimus may substitute or complement surgical therapy. Furthermore, also disadvantages of a possible treatment with sirolimus have to be considered including stomatitis and immunosuppression as also experienced in our patients. Furthermore, our clinical follow-up is limited and a longer follow up time is needed to assess long-term outcomes and potential complications. Nevertheless, further investigation is needed to understand the pathogenesis of lymphangiomatosis to establish further therapeutic approaches. In order to obtain further insights into clinical characteristics and to investigate long-term results of therapy in a larger population, a patient registry of lymphangiomatosis should be implemented.

In conclusion, we report here the largest series of an ultrarare disease, pulmonary lymphangiomatosis giving new insights into clinical characteristics and outcome. Furthermore, the reported data support a potential efficacy and effectiveness of sirolimus in the treatment of pulmonary lymphangiomatosis.

## Supplementary Information

Supplementary Material 1. Case reports on pulmonary lymphangiomatosis. List of case reports (citations) on pulmonary lymphangiomatosis and pulmonary involvement of lymphangiomatosis respectively in PubMed Central® (PMC) 1/1/1984–21/10/2024.

## Data Availability

No datasets were generated or analysed during the current study.
