# Supplementary material for: Pulmonary lymphangiomatosis: insights into an ultra-rare disease
Source: Respir Res. 2024 Nov 26;25:416. doi: 10.1186/s12931-024-03040-5 (PMC11600747; doi:10.1186/s12931-024-03040-5)
Supplement: Supplementary file 1 — Supplementary Material 1. Case reports on pulmonary lymphangiomatosis. List of case reports (citations) on pulmonary lymphangiomatosis and pulmonary involvement of lymphangiomatosis respectively in PubMed Central® (PMC) 1/1/1984–21/10/2024. [file 12931_2024_3040_MOESM1_ESM.docx]

**Case reports on „pulmonary lymphangiomatosis“**

**PubMed Central® (PMC) 1/1/1984-21/10/2024**

1. Biscotto I, Rodrigues RS, Forny DN, Barreto MM, Marchiori E. Diffuse pulmonary lymphangiomatosis. J Bras Pneumol. 2019 Sep 16;45(5):e20180412. doi: 10.1590/1806-3713/e20180412. PMID: 31531617; PMCID: PMC7247761.
2. Kadakia KC, Patel SM, Yi ES, Limper AH. Diffuse pulmonary lymphangiomatosis. Can Respir J. 2013 Jan-Feb;20(1):52-4. doi: 10.1155/2013/971350. PMID: 23457676; PMCID: PMC3628648.
3. Ernotte C, Médart L, Collignon L. Diffuse Pulmonary Lymphangiomatosis. J Belg Soc Radiol. 2018 Oct 4;102(1):64. doi: 10.5334/jbsr.1603. PMID: 30324183; PMCID: PMC6183323.
4. Onyeforo E, Barnett A, Zagami D, Deller D, Feather I. Diffuse pulmonary lymphangiomatosis treated with bevacizumab. Respirol Case Rep. 2018 Nov 28;7(1):e00384. doi: 10.1002/rcr2.384. PMID: 30510764; PMCID: PMC6260917.
5. Yu W, Mi L, Cong J, Cheng W, Chen Y, Gong H. Diffuse pulmonary lymphangiomatosis: A rare case report in an adult. Medicine (Baltimore). 2019 Oct;98(43):e17349. doi: 10.1097/MD.0000000000017349. PMID: 31651839; PMCID: PMC6824776.
6. Gurskytė V, Zeleckienė I, Maskoliūnaitė V, Mickys U, Šileikienė V. Successful treatment of diffuse pulmonary lymphangiomatosis with sirolimus. Respir Med Case Rep. 2020 Feb 1;29:101014. doi: 10.1016/j.rmcr.2020.101014. PMID: 32071853; PMCID: PMC7013175.
7. Moreno RP, Hernández Y, Garrido P, Camargo Vargas B, Hernández A, Faín J, Seligra C, Topp P, Maffey A, Cardigni G. Linfangiomatosis pulmonar difusa con compromiso pleural y pericárdico. Reporte de un caso pediátrico [Diffuse pulmonary lymphangiomatosis with pleural and pericardial involvement. Pediatric case report]. Arch Argent Pediatr. 2021 Jun;119(3):e264-e268. Spanish. doi: 10.5546/aap.2021.e264. PMID: 34033435.
8. Averyanov AV, Balionis OI, Divakova TI, Zabozlaev FG, Sotnikova AG. Severe Pulmonary Lymphedema in a Patient with Diffuse Pulmonary Lymphangiomatosis. Am J Respir Crit Care Med. 2019 Nov 1;200(9):e91-e92. doi: 10.1164/rccm.201901-0062IM. PMID: 31162933.
9. Salcedo Miranda D, Galvis JR, Téllez Rodríguez LJ, Garzón Ramírez JC, Ariza Traslaviña J. Diffuse pulmonary lymphangiomatosis as a differential diagnosis of anterior mediastinal mass. J Surg Case Rep. 2024 Sep 12;2024(9):rjae577. doi: 10.1093/jscr/rjae577. PMID: 39280023; PMCID: PMC11393567.
10. Caballero Y, Pérez D, Cano JR. Diffuse pulmonary lymphangiomatosis with mediastinal affectation. Arch Bronconeumol. 2011 Sep;47(9):474-5. English, Spanish. doi: 10.1016/j.arbres.2011.06.001. Epub 2011 Aug 6. PMID: 21821337.
11. Fang X, Huang Z, Zeng Y, Zhu X, Wang S, Yu X, Li X, Wu C, Yi X. Lymphangiomatosis involving the pulmonary and extrapulmonary lymph nodes and surrounding soft tissue: A rare case report. Medicine (Baltimore). 2017 Dec;96(49):e9032. doi: 10.1097/MD.0000000000009032. PMID: 29245295; PMCID: PMC5728910.
12. Swensen SJ, Hartman TE, Mayo JR, Colby TV, Tazelaar HD, Müller NL. Diffuse pulmonary lymphangiomatosis: CT findings. J Comput Assist Tomogr. 1995 May-Jun;19(3):348-52. doi: 10.1097/00004728-199505000-00002. PMID: 7790540.
13. Dimiene I, Bieksiene K, Zaveckiene J, Andrulis M, Optazaite DE, Vaguliene N, Zemaitis M, Miliauskas S. Effective Initial Treatment of Diffuse Pulmonary Lymphangiomatosis with Sirolimus and Propranolol: A Case Report. Medicina (Kaunas). 2021 Nov 29;57(12):1308. doi: 10.3390/medicina57121308. PMID: 34946253; PMCID: PMC8706407.
14. Shen QY, Nong GM, Gu YY. [Diffuse pulmonary lymphangiomatosis: two cases report]. Zhonghua Er Ke Za Zhi. 2016 Oct 2;54(10):781-782. Chinese. doi: 10.3760/cma.j.issn.0578-1310.2016.10.017. PMID: 27784485.
15. Rasalkar DD, Chu WC. Generalized cystic lymphangiomatosis. Pediatr Radiol. 2010 Dec;40 Suppl 1:S47. doi: 10.1007/s00247-010-1694-7. Epub 2010 Jun 24. PMID: 20574655.
16. Le HDT, Vo DS, Le DD, Dang CT, Nguyen Thanh T. Generalized lymphangiomatosis-A rare manifestation of lymphatic malformation. Radiol Case Rep. 2020 Nov 2;16(1):66-71. doi: 10.1016/j.radcr.2020.10.044. Erratum in: Radiol Case Rep. 2022 Jul 16;17(9):3450. doi: 10.1016/j.radcr.2022.06.054. PMID: 33193931; PMCID: PMC7642761.
17. Nagano N, Izumi S, Katsuno T, Iikura M, Miyazaki H, Igari T, Okafuji T, Sekihara K, Nagasaka S, Hojo M. A case of diffuse pulmonary lymphangiomatosis with a venous anomaly presenting with acute respiratory failure and hemoptysis. Respir Med Case Rep. 2020 Oct 7;31:101243. doi: 10.1016/j.rmcr.2020.101243. PMID: 33088708; PMCID: PMC7567044.
18. Nèji H, Hantous-Zannad S, Zidi A, Hamzaoui A, Mezni F, Baccouche I, Ben Miled-M'rad K. Pneumopathie interstitielle diffuse chez une enfant [Diffuse pulmonary lymphangiomatosis in a child]. Arch Pediatr. 2013 Nov;20(11):1234-1235. French. doi: 10.1016/j.arcped.2013.08.006. Epub 2013 Sep 27. PMID: 24080036.
19. de Lima AS, Martynychen MG, Florêncio RT, Rabello LM, de Barros JA, Escuissato DL. Pulmonary lymphangiomatosis: a report of two cases. J Bras Pneumol. 2007 Mar-Apr;33(2):229-33. English, Portuguese. doi: 10.1590/s1806-37132007000200020. PMID: 17724545.
20. Zhao J, Wu R, Gu Y. Pathology analysis of a rare case of diffuse pulmonary lymphangiomatosis. Ann Transl Med. 2016 Mar;4(6):114. doi: 10.21037/atm.2016.03.30. PMID: 27127767; PMCID: PMC4828737.
21. Zheng G, Tang H, Su R, Liang Y, He Z, Zhang J, Deng J, Bai J, Zhong X. A gene missense mutation in diffuse pulmonary lymphangiomatosis with thrombocytopenia: A case report. Medicine (Baltimore). 2020 Sep 25;99(39):e21941. doi: 10.1097/MD.0000000000021941. PMID: 32991402; PMCID: PMC7523817.
22. Tran D, Fallat ME, Buchino JJ. Lymphangiomatosis: a case report. South Med J. 2005 Jun;98(6):669-71. doi: 10.1097/01.SMJ.0000145308.72031.3E. PMID: 16004177.
23. Aman J, Thunnissen E, Paul MA, van Nieuw Amerongen GP, Vonk-Noordegraaf A. Successful treatment of diffuse pulmonary lymphangiomatosis with bevacizumab. Ann Intern Med. 2012 Jun 5;156(11):839-40. doi: 10.7326/0003-4819-156-11-201206050-00016. PMID: 22665821.
24. Takahashi K, Takahashi H, Maeda K, Homma S, Uekusa T, Dambara T, Kira S. An adult case of lymphangiomatosis of the mediastinum, pulmonary interstitium and retroperitoneum complicated by chronic disseminated intravascular coagulation. Eur Respir J. 1995 Oct;8(10):1799-802. doi: 10.1183/09031936.95.08101799. PMID: 8586141.
25. Riazuddin M, Farouk NI, Ali SS, Butt MI, Arabi TZ, Sabbah BN, Ali MS, Alkattan K. Pleural effusion due to lymphangiomatosis and the role of sirolimus: A case report. Ann Med Surg (Lond). 2023 Oct 5;85(12):6178-6181. doi: 10.1097/MS9.0000000000001384. PMID: 38098585; PMCID: PMC10718374.
26. Kinnier CV, Eu JP, Davis RD, Howell DN, Sheets J, Palmer SM. Successful bilateral lung transplantation for lymphangiomatosis. Am J Transplant. 2008 Sep;8(9):1946-50. doi: 10.1111/j.1600-6143.2008.02340.x. Epub 2008 Jul 28. PMID: 18671675; PMCID: PMC3732029.
27. Chen YL, Lee CC, Yeh ML, Lee JS, Sung TC. Generalized lymphangiomatosis presenting as cardiomegaly. J Formos Med Assoc. 2007 Mar;106(3 Suppl):S10-4. doi: 10.1016/s0929-6646(09)60359-4. PMID: 17493902.
28. Libby LJ, Narula N, Fernandes H, Gruden JF, Wolf DJ, Libby DM. Imatinib Treatment of Lymphangiomatosis (Generalized Lymphatic Anomaly). J Natl Compr Canc Netw. 2016 Apr;14(4):383-6. doi: 10.6004/jnccn.2016.0045. PMID: 27059187.
29. Ayadi-Kaddour A, Cherif J, Mlika M, Béji M, El Mezni F. Lymphangiomatose pulmonaire révélée par un syndrome interstitiel chez une adolescente [Pulmonary lymphangiomatosis revealed by an interstitial syndrome in a young adult]. Tunis Med. 2009 Apr;87(4):289-91. French. PMID: 19835289.
30. Ozeki M, Fukao T, Kondo N. Propranolol for intractable diffuse lymphangiomatosis. N Engl J Med. 2011 Apr 7;364(14):1380-2. doi: 10.1056/NEJMc1013217. PMID: 21470038.
31. Sun X, Lu C, Huang Z, Xu J, Zhu H, Yang S, Chen D. Diagnosis and treatment of diffuse pulmonary lymphangioma in children: A case report. Exp Ther Med. 2023 Mar 7;25(4):175. doi: 10.3892/etm.2023.11874. PMID: 37006871; PMCID: PMC10061045.
32. Zhang J, Jin H, Wang Y, Bai C, Han Y. A case of diffuse pulmonary lymphangiomatosis with unilateral lung invasion. Oxf Med Case Reports. 2015 Oct 27;2015(10):346-8. doi: 10.1093/omcr/omv059. PMID: 26512334; PMCID: PMC4622182.
33. Chira RI, Florea A, Pârvu D, Chira A, Crişan D, Popovici B. Transthoracic ultrasound: an essential diagnostic tool in a very rare case of thoracic lymphangiomatosis. Med Ultrason. 2021 Aug 11;23(3):361-363. doi: 10.11152/mu-2499. Epub 2020 May 28. PMID: 32905574.
34. Hangul M, Kose M, Ozcan A, Unal E. Propranolol treatment for chylothorax due to diffuse lymphangiomatosis. Pediatr Blood Cancer. 2019 May;66(5):e27592. doi: 10.1002/pbc.27592. Epub 2019 Jan 4. PMID: 30609288.
35. Harnisch E, Sukhai R, Oudesluys-Murphy AM. Serious complications of pulmonary biopsy in a boy with chylopericardium and suspected pulmonary lymphangiomatosis. BMJ Case Rep. 2010 May 6;2010:bcr08.2009.2206. doi: 10.1136/bcr.08.2009.2206. PMID: 22736725; PMCID: PMC3047284.
36. Bermejo Casero EJ, Mongil Poce R, Arrabal Sánchez R, Fernández de Rota Avecilla A, Benítez Doménech A, Fernández Bermúdez JL. Linfangiomatosis torácica difusa: diagnóstico y tratamiento [Diffuse thoracic lymphangiomatosis: diagnosis and treatment]. Arch Bronconeumol. 2004 Dec;40(12):599-601. Spanish. PMID: 15574275.
37. Putta T, Irodi A, Thangakunam B, Oliver A, Gunasingam R. Young patient with generalized lymphangiomatosis: Differentiating the differential. Indian J Radiol Imaging. 2016 Jul-Sep;26(3):411-415. doi: 10.4103/0971-3026.190416. PMID: 27857472; PMCID: PMC5036344.
38. Mehrnahad M, Kord A, Rezaei Z, Kord R. Late diagnosis of generalized lymphangiomatosis in a woman presenting with respiratory distress. Radiol Case Rep. 2020 Jun 9;15(8):1189-1193. doi: 10.1016/j.radcr.2020.05.021. PMID: 32550956; PMCID: PMC7292890.
39. Ramani P, Shah A. Lymphangiomatosis. Histologic and immunohistochemical analysis of four cases. Am J Surg Pathol. 1993 Apr;17(4):329-35. PMID: 8494102.
40. Maurac A, Debray MP, Crestani B, Taillé C. Thoracic involvement of diffuse lymphangiomatosis successfully treated with sildenafil. BMJ Case Rep. 2019 Apr 23;12(4):e228523. doi: 10.1136/bcr-2018-228523. PMID: 31015245; PMCID: PMC6510121.
41. Yekeler E, Dursun M, Yildirim A, Tunaci M. Diffuse pulmonary lymphangiomatosis: imaging findings. Diagn Interv Radiol. 2005 Mar;11(1):31-4. PMID: 15795841.
42. Nair LG, Kurtz CP. Lymphangiomatosis presenting with bronchial cast formation. Thorax. 1996 Jul;51(7):765-6. doi: 10.1136/thx.51.7.765. PMID: 8882088; PMCID: PMC472504.
43. Oztunç F, Koca B, Adaletli I. Generalised lymphangiomatosis in an 8-year-old girl who presented with cardiomegaly. Cardiol Young. 2011 Aug;21(4):465-7. doi: 10.1017/S1047951111000205. Epub 2011 Mar 2. PMID: 21362206.
44. Minakata T, Suzuki T, Kamio Y, Kadokura M, Himuro N, Takei H. A Case of Lymphangiomatosis With Infected Lymphangiomas Effectively Treated by Thoracoscopic Debridement and Drainage. Chest. 2020 Nov;158(5):e221-e224. doi: 10.1016/j.chest.2020.06.048. PMID: 33160540.
45. Liu Y, Sui X, Chen K, Li J, Yang F, Ho AK, Wang J. Thoracic lymphangiomatosis: report of 3 patients with different presentations. Ann Thorac Surg. 2012 Dec;94(6):2111-3. doi: 10.1016/j.athoracsur.2012.04.056. PMID: 23176925.
46. Glöckler M, Severin T, Arnold R, Greiner P, Schwab KO, Uhl M, Schlensak C, Rössler J, Dittrich S. First description of three patients with multifocal lymphangiomatosis and protein-losing enteropathy following palliation of complex congenital heart disease with total cavo-pulmonary connection. Pediatr Cardiol. 2008 Jul;29(4):771-4. doi: 10.1007/s00246-007-9194-8. Epub 2008 Jan 11. PMID: 18188635.
47. Yang DH, Goo HW. Generalized lymphangiomatosis: radiologic findings in three pediatric patients. Korean J Radiol. 2006 Oct-Dec;7(4):287-91. doi: 10.3348/kjr.2006.7.4.287. PMID: 17143033; PMCID: PMC2667616.
48. Boland JM, Tazelaar HD, Colby TV, Leslie KO, Hartman TE, Yi ES. Diffuse pulmonary lymphatic disease presenting as interstitial lung disease in adulthood: report of 3 cases. Am J Surg Pathol. 2012 Oct;36(10):1548-54. doi: 10.1097/PAS.0b013e31825eae67. Erratum in: Am J Surg Pathol. 2013 May;37(5):779. PMID: 22982897.
49. Tamay Z, Saribeyoglu E, Ones U, Anak S, Güler N, Bilgic B, Yilmazbayhan D, Gun F. Diffuse thoracic lymphangiomatosis with disseminated intravascular coagulation in a child. J Pediatr Hematol Oncol. 2005 Dec;27(12):685-7. doi: 10.1097/01.mph.0000193476.14493.06. PMID: 16344679.
50. Steinacher I, Lamprecht B, Lobendanz M, Zoller H, Dartevelle P, Fadel E, Studnicka M. Successful surgical treatment of thoracic multiorgan lymphangiomatosis. Wien Klin Wochenschr. 2009;121(19-20):644-7. doi: 10.1007/s00508-009-1249-9. PMID: 19921132.
51. Liu JR, Shen WB, Wen Z, An R, Zhou CJ, Zhao SY. [Clinical analysis of two cases with diffuse pulmonary lymphatic disease]. Zhonghua Er Ke Za Zhi. 2016 May;54(5):360-4. Chinese. doi: 10.3760/cma.j.issn.0578-1310.2016.05.010. PMID: 27143078.
52. Laverdière C, David M, Dubois J, Russo P, Hershon L, Lapierre JG. Improvement of disseminated lymphangiomatosis with recombinant interferon therapy. Pediatr Pulmonol. 2000 Apr;29(4):321-4. doi: 10.1002/(sici)1099-0496(200004)29:4<321::aid-ppul13>3.0.co;2-c. PMID: 10738021.
53. Fukahori S, Tsuru T, Asagiri K, Nakamizo H, Asakawa T, Tanaka H, Tanaka Y, Akiba J, Yano H, Yagi M. Thoracic lymphangiomatosis with massive chylothorax after a tumor biopsy and with disseminated intravenous coagulation--lymphoscintigraphy, an alternative minimally invasive imaging technique: report of a case. Surg Today. 2011 Jul;41(7):978-82. doi: 10.1007/s00595-010-4383-0. Epub 2011 Jul 12. PMID: 21748615.
54. Kandil A, Rostom AY, Mourad WA, Khafaga Y, Gershuny AR, el-Hosseiny G. Successful control of extensive thoracic lymphangiomatosis by irradiation. Clin Oncol (R Coll Radiol). 1997;9(6):407-11. doi: 10.1016/s0936-6555(97)80140-9. PMID: 9498881.
55. Cogorno-Wasylkowski L, Martínez-Pérez E, Carvajal-Buitrago DF, Vázquez-Rodríguez G, Guinda-Sevillano CE. Uncommon renal masses: Perirenal extramedullary hematopoiesis and multiple lymphangiomatosis with a perirenal lymphangioma. Arch Esp Urol. 2014 Dec;67(10):848-52. English, Spanish. PMID: 25582904.
56. Cogorno-Wasylkowski L, Martínez-Pérez E, Carvajal-Buitrago DF, Vázquez-Rodríguez G, Guinda-Sevillano CE. Uncommon renal masses: Perirenal extramedullary hematopoiesis and multiple lymphangiomatosis with a perirenal lymphangioma. Arch Esp Urol. 2014 Dec;67(10):848-52. English, Spanish. PMID: 25582904.
57. Villegas Fernández FR, Callol Sánchez L, Moreno Muro M, Coca Menchero S, García Pérez C, Gómez de Terreros FJ. Linfangiomiomatosis pulmonar asociada con angiomiolipoma renal. Presentación de un caso y revisión de la literatura [Pulmonary lymphangiomatosis associated with renal angiomyolipoma. Presentation of a case and review of the literature]. Rev Clin Esp. 1992 Jul;191(3):144-7. Spanish. PMID: 1502405.
58. Swank DW, Hepper NG, Folkert KE, Colby TV. Intrathoracic lymphangiomatosis mimicking lymphangioleiomyomatosis in a young woman. Mayo Clin Proc. 1989 Oct;64(10):1264-8. doi: 10.1016/s0025-6196(12)61289-0. PMID: 2593716.
59. Datz C, Graziadei IW, Dietze O, Jaschke W, Königsrainer A, Sandhofer F, Margreiter R. Massive progression of diffuse hepatic lymphangiomatosis after liver resection and rapid deterioration after liver transplantation. Am J Gastroenterol. 2001 Apr;96(4):1278-81. doi: 10.1111/j.1572-0241.2001.03712.x. PMID: 11316184.
60. Nakajima J, Goto A, Takamoto S, Murakawa T, Fukami T, Kusakabe M. Invasive lymphangioma of the lung manifesting as a large pulmonary mass with hemoptysis: report of a case. Surg Today. 2007;37(5):418-22. doi: 10.1007/s00595-006-3412-5. Epub 2007 Apr 30. PMID: 17468825.
61. Shah AR, Dinwiddie R, Woolf D, Ramani R, Higgins JN, Matthew DJ. Generalized lymphangiomatosis and chylothorax in the pediatric age group. Pediatr Pulmonol. 1992 Oct;14(2):126-30. doi: 10.1002/ppul.1950140211. PMID: 1437350.
62. Bhatti MA, Ferrante JW, Gielchinsky I, Norman JC. Pleuropulmonary and skeletal lymphangiomatosis with chylothorax and chylopericardium. Ann Thorac Surg. 1985 Oct;40(4):398-401. doi: 10.1016/s0003-4975(10)60078-1. PMID: 4051622.
63. Patton DF, Kaye R, Dickman P, Blatt J. Partial splenic embolization for treatment of disseminated intravascular coagulation in lymphangiomatosis. J Pediatr. 1998 Jun;132(6):1057-60. doi: 10.1016/s0022-3476(98)70412-3. PMID: 9627607.
64. Abou Hamdan K, Chabot F, Capron F, Debelle L, Fery M, Polu JM. Lymphangiomatose pulmonaire diffuse chez un adulte jeune [Diffuse pulmonary lymphangiomatosis in a young adult]. Rev Mal Respir. 2001 Sep;18(4 Pt 1):436-9. French. PMID: 11547254.
65. Ozturk A, Yousem DM. Magnetic resonance imaging findings in diffuse lymphangiomatosis: neuroradiological manifestations. Acta Radiol. 2007 Jun;48(5):560-4. doi: 10.1080/02841850701352038. PMID: 17520434.
66. Makarian RS, Mirea O, Verhamme P, Smeyers KM, Berkmans E, Raicea V, Berceanu M, Van Raemdonck D, Ceulemans LJ. Pericardial fenestration and thoracic duct ligation for treatment of chylopericardium as first symptom of underlying generalized lymphatic anomaly: a case report. Acta Chir Belg. 2024 Sep 26:1-4. doi: 10.1080/00015458.2024.2406606. Epub ahead of print. PMID: 39324580.
67. Higgins JN, Shah AR, Dicks-Mireaux CF, Conry BG. Case report: computed tomography of generalized lymphangiomatosis and chylothorax. Br J Radiol. 1993 Dec;66(792):1189-92. doi: 10.1259/0007-1285-66-792-1189. PMID: 8293266.
68. Hamamoto R, Nishimori A, Izaki T, Okumura K, Ohshiro H, Yamamoto H, Inomata Y. Drainage of subcutaneous lymphatic fluid for the management of respiratory distress in a case of generalized lymphangiectasia in an infant. Pediatr Surg Int. 2003 May;19(3):204-6. doi: 10.1007/s00383-002-0919-8. Epub 2003 May 27. PMID: 12768313.
69. Kothari SS, Sharma S, Bhatt K, Ray R, Bakhshi S, Chowdhury U. Recurrent hemorrhagic pericardial effusion in a child due to diffuse lymphangiohemangiomatosis: a case report. J Med Case Rep. 2010 Feb 22;4:62. doi: 10.1186/1752-1947-4-62. PMID: 20170552; PMCID: PMC2831910.
70. Wiatr E, Langfort R, Orłowski T, Grudny J, Gawryluk D, Kupis W, Usiekniewicz J, Oniszh K, Burakowska B, Roszkowski K. Chłonkotok opłucnowy w przebiegu naczyniakowatości limfatycznej płuc [Chylothorax in patients with diffuse pulmonary lymphangiomatosis]. Pneumonol Alergol Pol. 2006;74(2):209-15. Polish. PMID: 17269371.
71. Desideri V, Rottoli ML, Comin CE, Colagrande S. A proposito di un caso di linfangiomatosi polmonare: valutazione con Tomografia Computerizzata ad Alta Risoluzione (TCAR) e correlazioni anatomo-radiologiche [A case of pulmonary lymphangiomatosis: high resolution computed tomography (HRCT) evaluation and correlation with anatomo-radiological findings]. Radiol Med. 2001 May;101(5):386-8. Italian. PMID: 11438793.
